# Left-sided Catamenial Pneumothorax: A Rare Clinical Entity

**DOI:** 10.7759/cureus.2567

**Published:** 2018-05-02

**Authors:** Naureen Narula, Sam Ngu, Akshay Avula, Wissam Mansour, Michel Chalhoub

**Affiliations:** 1 Internal Medicine, Staten Island University Hospital, Staten Island, USA; 2 Pulmonary and Critical Care Medicine, Staten Island University Hospital, Northwell Health, Staten Island, USA; 3 Pulmonary Medicine and Critical Care, Staten Island University Hospital, Northwell Health, Staten Island, USA

**Keywords:** catamenial pneumothorax

## Abstract

Catamenial pneumothorax (CP) is an extremely rare pulmonary pathology seen in women of reproductive age, typically occurring within 72 hours from the onset of menstrual bleeding. Multiple theories have been proposed to explain the etiopathogenesis of CP; however, the exact underlying mechanism remains elusive. More than 90% of reported cases in the literature describe a right-sided presentation of pneumothorax. In this case report, we describe a rare left-sided presentation of CP and discuss the current literature on underlying etiopathogenesis, diagnostics, and available therapeutic modalities for managing this rare clinical entity.

## Introduction

The presence of endometrial tissue outside the uterine cavity is termed endometriosis while its presence inside the thoracic cavity is known as thoracic endometriosis [1-2]. Thoracic endometriosis syndrome includes four distinct clinical presentations: catamenial pneumothorax (CP), catamenial hemothorax, hemoptysis, and pulmonary nodules [3]. CP is a rare form of spontaneous pneumothorax (PTX) that occurs predominantly in women of reproductive age, typically within 72 hours from the onset of menstrual bleeding [1-3]. In 1958, Maurer et al. described the first case of recurrent PTX associated with concomitant menstruation [4]. Later in 1972, Lillington et al. [5] successfully coined the definition of CP. Current literature estimates suggest that CP accounts for up to one-third of surgically treated cases of PTX in women. It is the most common manifestation of thoracic endometriosis syndrome (TES). More than 90% of cases report a right-sided predilection for PTX. In this report, we describe an unusual presentation of left-sided CP and discuss the underlying etiopathogenesis and the available diagnostic and therapeutic modalities for managing CP.

## Case presentation

**Figure 1:**
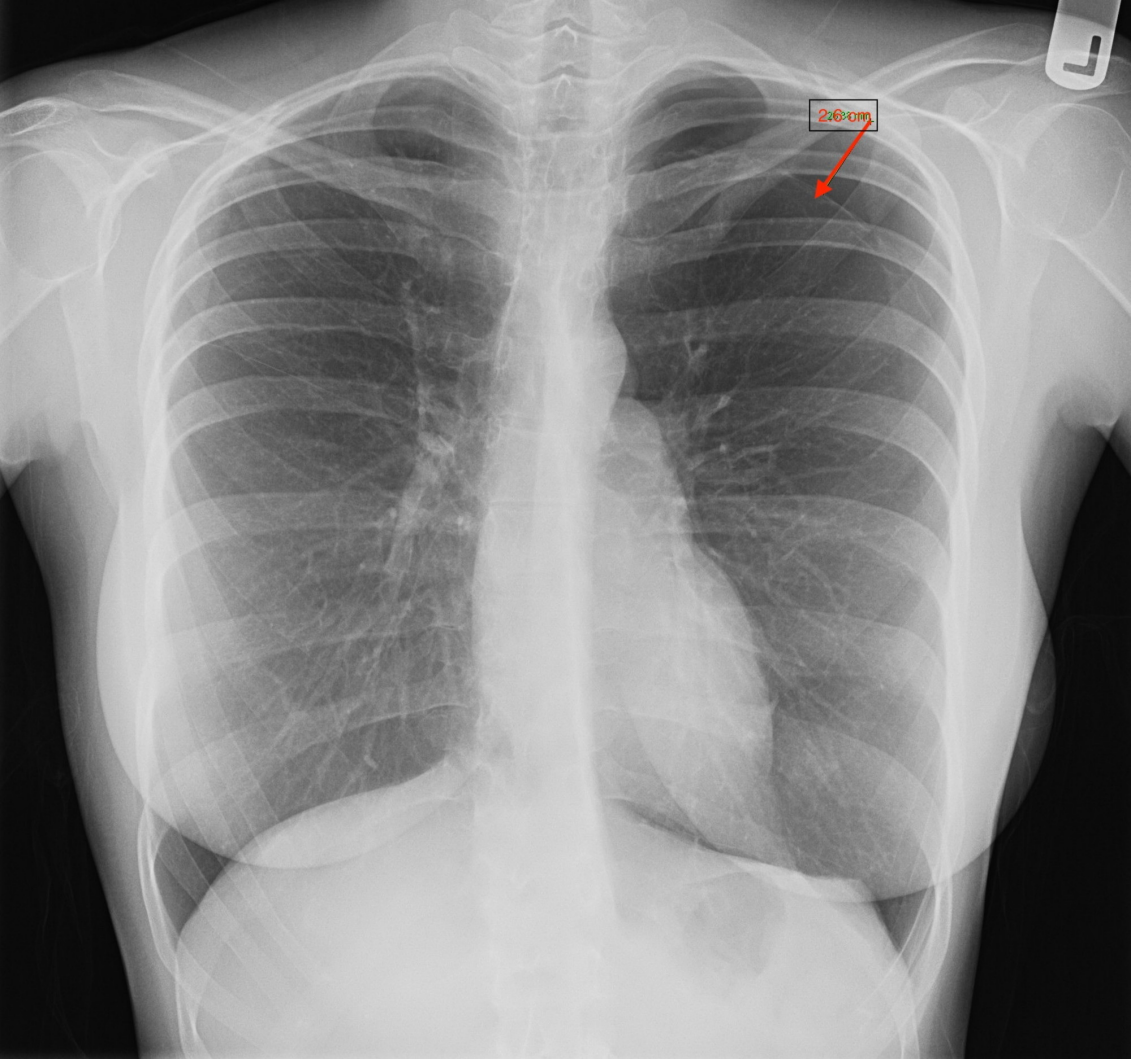
Chest x-ray Chest x-ray (CXR) showing a moderate-sized left pneumothorax with a 2.6 cm gap.

A previously healthy, 24-year-old female presented to the emergency department (ED) with a one-day history of shortness of breath (SOB) and left-sided pleuritic chest pain. She complained experiencing progressively worsening dyspnea with physical activity. SOB was associated with left-sided chest pain described as stabbing in character, Th pain rated 6/10 in intensity on the pain scale and was non-radiating. No pertinent medical, surgical, or social history was noted. She denied a recent history of trauma. The patient was on the second day of her menstrual cycle. In the ED, her vitals showed a blood pressure of 125/70 mmHg, a heart rate of 102 beats/minute and a respiratory rate of 19 breaths/min. She was afebrile and was maintaining an oxygen saturation of 99% on room air. On physical examination, she had diminished breath sounds on the left side. Chest x-ray (CXR) showed a moderate-sized left PTX with a 2.6 cm gap but without a mediastinal shift (Figure 1).

Needle aspiration with the placement of a left-sided pigtail catheter was performed immediately. Blood work and basic metabolic chemistry were within the normal limits. Venous blood gas was grossly normal with the exception of lactic acidosis (2.8 mmol/L). For her pain, the patient received analgesics and symptomatic improvement was noted. A repeat CXR showed a decrease in the size of the PTX from 2.6 cm to 1.9 cm. A consecutive CXR over the next two days showed significant improvement. A complete resolution of PTX was noted eventually. Consultants from pulmonary and obstetrics/gynecology confirmed the clinical diagnosis of catamenial-pneumothorax possibly associated with thoracic endometriosis. She was eventually discharged on day five of hospitalization on an oral contraceptive medication containing ethinyl estradiol and levonorgestrel.

## Discussion

Thoracic endometriosis syndrome (TES) is characterized by the presence of endometrial tissue in or around the lung, and it consists of four distinct clinical presentations: catamenial pneumothorax (CP), catamenial hemothorax, hemoptysis, and pulmonary nodules [3]. CP consists of 30%-50% of all cases of thoracic endometriosis syndrome (TES) [1]. A diagnosis of CP requires a high degree of clinical suspicion relating pulmonary symptoms temporally to the phase of menstrual cycle. According to the widely accepted endometrial implant theory, endometrial tissues in the thorax rapidly proliferate and, subsequently, lead to a necrosis of the tissue, causing chest pain along with dyspnea, dry cough, pneumothorax, and, sometimes, hemoptysis [1-2]. Despite a temporal relationship with menstruation, the exact pathogenesis of catamenial pneumothorax remains elusive. Multiple theories have been postulated to describe the etiopathogenesis. The theory of coelomic metaplasia, prostaglandin theory, theory of retrograde menstruation, passage of air through the diaphragm, and lymphatic/vascular microembolization are some of the few widely accepted hypothesis [1,3,6]. Pathogenesis may be multifactorial, as described by Bricelj K et al. In this study, endometrial implants were seen on video-assisted thoracoscopic surgery (VATS) in 59.3% of all patients diagnosed with CP, whereas diaphragmatic defects ranging 1 mm to 10 mm were reported in 57.0% of patients [7]. The possible explanation for a right-sided predilection may be related to the clockwise movement of endometrial tissue within the peritoneal fluid from the pelvis along the right paracolic gutter to the subphrenic space. Two forces contribute to this movement: the falciform ligament preventing the movement of endometrial tissue to the left and the intraperitoneal pressure variations during respiration causing the right hemidiaphragm to contract against liver. This generates a piston-like effect, which potentiates the endometrial tissue movement across the right hemidiaphragm [8].

CXR and a chest computed tomography (CT) scan have a limited role in delineating the endometrial source of origin of pneumothorax. CT chest may show associated pneumoperitoneum or diaphragmatic endometrial implants. Chest magnetic resonance imaging (MRI) may help in identifying hyperintense diaphragmatic and pleural endometriosis lesions on T1- and T2-weighted images [1]. However, direct visualization with VATS and histopathologic confirmation is considered the gold standard diagnostic modality [9]. Bagan et al. reported elevated serum levels of CA-125 as a possible diagnostic aid; however, CA-125 levels lack specificity; thus, routine monitoring of the levels is not recommended [10]. While there are no prospective trials comparing various management strategies, the treatment modalities for CP remain similar to that for endometriosis. Hormonal treatment is the cornerstone medical management therapy. It blocks feedback to the endometrial tissue and, therefore, prevents further dissemination. Oral contraceptives, danazol, and gonadotropin-releasing hormone (GnRH) analogs are commonly used therapeutics for managing CP [11]. Recently, dienogest - a synthetic oral progestogen, has shown potential benefits in preventing the recurrence of CP [12]. The surgical options include hysterectomy with bilateral salpingo-oophorectomy. Surgery is considered a definitive treatment and is usually reserved for severe refractory cases [6]. VATS-assisted resection of ectopic endometrial implants and the repair of diaphragmatic and pleural defects can theoretically lead to disease resolution. However, several cases of disease recurrence have been reported despite a repair of diaphragmatic defects [7]. A multidisciplinary approach collaborating pulmonary and gynecological consultation is considered ideal as the possibility of concomitant uterine endometriosis is fairly high in patients with CP.

Table 1 summarizes various theories explaining the etiopathogenesis of CP.

**Table 1:** Theories explaining the etiopathogenesis of catamenial pneumothorax

Theories	Explanation	References
Prostaglandin theory	High circulating levels of prostaglandin F2 during menstruation may independently cause vasoconstriction and bronchospasm of endometrial implants, leading to alveolar rupture particularly in pre-existing bullae or blebs.	[13-18]
Coelomic-metaplasia	Tissue of coelomic origin, such as the pleura and the peritoneum, can differentiate into endometrial cells in response to estrogen stimulus. This explains the presence of endometrial tissue in patients without a uterus, including men on prolonged estrogen therapy.	[3,13-18]
Diaphragmatic theory of air passage	Sloughing of endometrial tissues implants within the pleura and diaphragm introduces fenestrations and defects that allows for the passage of air into the thorax, resulting in pneumothorax. Air supposedly flows into the peritoneum via the fallopian tubes due to the absence of a cervical mucus plug during the menstrual cycle.	[3,13-18]
Retrograde menstruation (migration theory)	The clockwise directionality of peritoneal flow from the right paracolic gutter in combination with retrograde menstruation to influence the implantation of ectopic endometrial tissue onto the pleura and diaphragm, making it preferentially on the right side..	[3,13-18]
Lymphatic/vascular microembolization	Endometrial transplantation occurs through lymphatic/vascular microembolization. This explains the presence of both intrapulmonary and other extra-uterine sites of implantation.	[3,13-18]

## Conclusions

The diagnosis of CP requires a high degree of clinical suspicion. Diagnosis should be suspected in the presence of clinical signs of respiratory distress correlating temporaly with the menstrual cycle. Clinicians should not exclude diagnosis in left-sided presentations. The treatment may be either medical and/or surgical with the primary aim of avoiding disease recurrence. More longitudinal studies are required for formulating diagnostic and therapeutic guidelines for managing CP.
